# Complications and prognosis of primary thoracic and lumbar giant cell tumors treated by total tumor resection

**DOI:** 10.1186/s12891-023-06347-4

**Published:** 2023-04-12

**Authors:** Jiacheng Liu, Panpan Hu, Hua Zhou, Ben Wang, Xiaoguang Liu, Fengliang Wu, Yan Li, Xiao Liu, Lei Dang, Yanchao Tang, Zihe Li, Zhongjun Liu, Feng Wei

**Affiliations:** Department of Orthopedics and Beijing Key Laboratory of Spinal Disease Research, Peking University Third Hospital, 49 North Garden Rd, Haidian District, Beijing, 100191 China

**Keywords:** Spinal giant cell tumor, En bloc resection, Complication, Recurrence

## Abstract

**Background:**

Spinal giant cell tumor (SGCT) is a relatively rare primary tumor. En bloc resection is the preferred surgical procedure for it due to its aggressiveness, meanwhile leading to more complications. We reported the characteristics of perioperative complications and local control of total tumor resection including en bloc resection and piecemeal resection for primary thoracic and lumbar spinal giant cell tumors in a single center over 10 years.

**Methods:**

This is a retrospective cross-sectional and cohort study. Forty-one consecutive patients with SGCTs who underwent total tumor resection from 2010 to 2020 at our institution and were followed up for at least 24 months were reviewed. Surgery data, complication characteristics and local tumor control were collected and compared by different surgical procedure.

**Results:**

Forty-one patients were included, consisting of 18 males and 23 females, with a mean age of 34.2 years. Thirty-one had thoracic vertebra lesions, and 10 had lumbar vertebra lesions. Thirty-five patients were primary cases, and 6 patients were recurrent cases. Eighteen patients were treated by total en bloc spondylectomy (TES), 12 patients underwent en bloc resection according to WBB surgical system, and 11 patients underwent piecemeal resection. The average surgical time was 498 min, and the mean estimated blood loss was 2145 ml. A total of 58 complications were recorded, and 30 patients (73.2%) had at least one perioperative complication. All patients were followed up after surgery for at least 2 years. A total of 6 cases had postoperative internal fixation failure, and 4 cases presented local tumor recurrence (9.8%).

**Conclusions:**

Although the surgical technique is difficult and accompanied by a high rate of perioperative complications, en bloc resection can achieve favorable local control in SGCT. When it is too difficult to complete en bloc resection, thoroughly piecemeal resection without residual is also acceptable, given the relatively low recurrence rate.

## Introduction

Giant cell tumor (GCT) is a primary bone tumor. This locally aggressive tumor is more commonly seen in the epiphysis of the long diaphysis and in the spine, accounting for approximately 5% of all primary bone tumors [1]. Spinal giant cell tumor (SGCT) is a relatively rare primary tumor, accounting for approximately 1.4-9.4% of primary tumors of the spine, and is more common in females than in males [2]. Curettage can lead to a high local recurrence rate due to its aggressiveness and high local recurrence rate [3]; Denosumab is a monoclonal antibody against RANKL, which can specifically block the binding between RANKL and RANK, thereby inhibiting the formation, differentiation and activation of osteoclasts, reducing bone resorption, and achieving therapeutic effects [4]. Denosumab is mainly used in the treatment of refractory, recurrent or metastatic giant cell tumors of bone [5]. Considering the possibility of tumor recurrence after drug withdrawal, long-term use is recommended. However, with the increase of treatment time, the risk of adverse events such as osteonecrosis of the jaw increases [6]. Therefore, en bloc resection is the preferred surgical procedure for SGCT [7]. There are two main methods of en bloc resection: ① total en bloc spondylectomy (TES) [8, 9]and ② en bloc resection according to the WBB (Weinstein, Boriani, Biagnini) surgical system [10, 11]. However, spinal tumor is adjacent to the spinal cord, nerve root, vertebral artery and other important structures. It is difficult and risky to achieve the goal of en bloc resection, bringing greater damage to surrounding structures, leading to higher incidence of complications compared with other spine surgeries. It has been reported that the complication rate of en bloc spinal tumor resection is 46.2–86.7% [12–17]. The purpose of this study was to investigate the characteristics of perioperative complications and local tumor control of en bloc resection for primary thoracic and lumbar spinal giant cell tumors in Peking University Third Hospital over 10 years.

## Method

### Patients’ recruitment

Patients diagnosed with primary thoracic and lumbar GCT by pathology who received total tumor resection at the Peking University Third Hospital from 2010 to 2020 were enrolled, including primary and recurrent tumor. Patients who underwent palliative surgery or did not undergo surgery during the same period were excluded.

### Surgical procedures

**Fig. 1:**
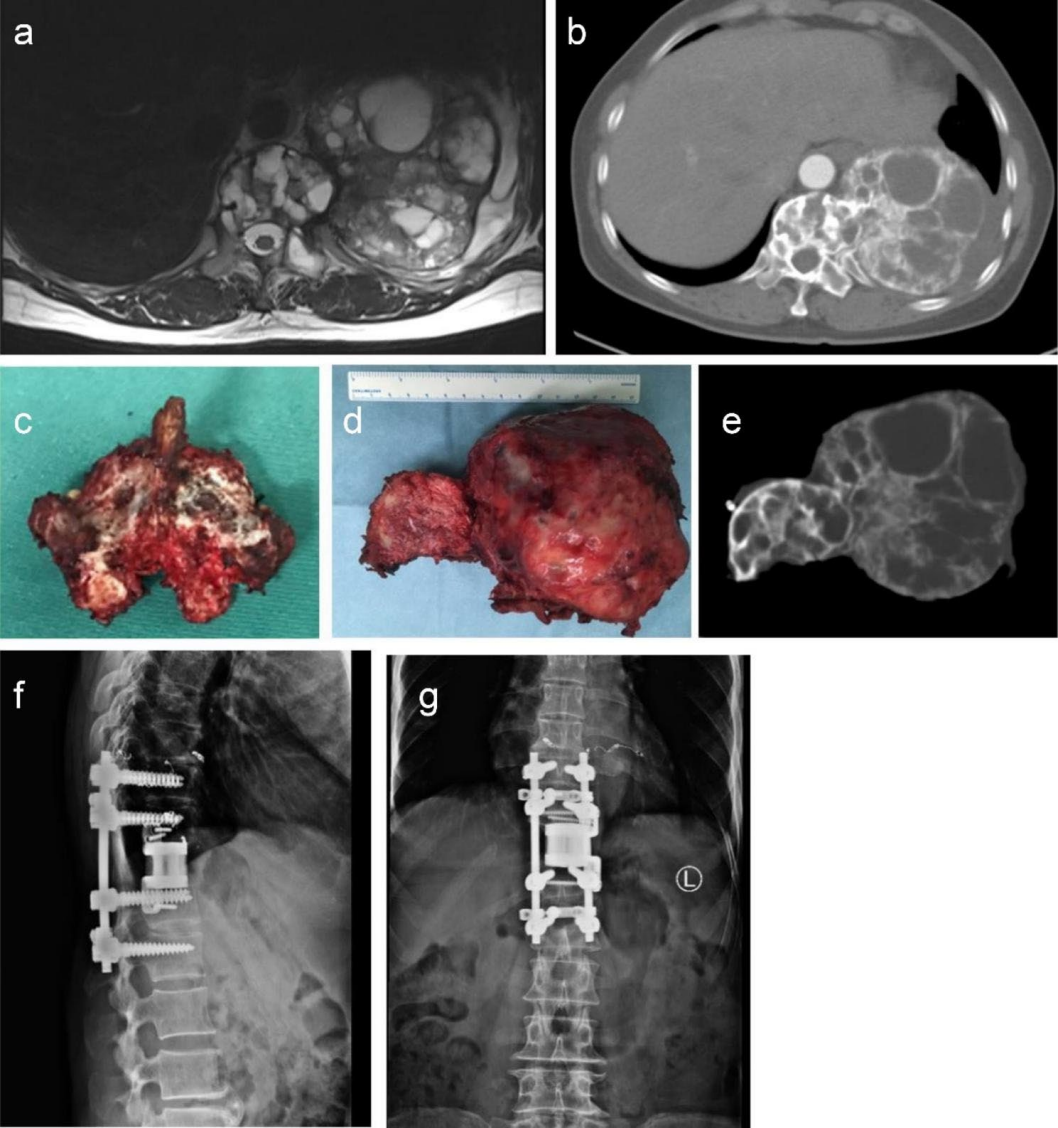
A 50-year-old female patient with T11 giant cell tumor, treated with denosumab for 3 months preoperatively, underwent TES surgery. Picture a and b were preoperative image of the tumor; picture c was the specimen of the posterior structure of the spine; picture d and e were specimen and image of the resected tumor and T11 vertebral body; picture f and g were postoperative image after 4 years

**Fig. 2:**
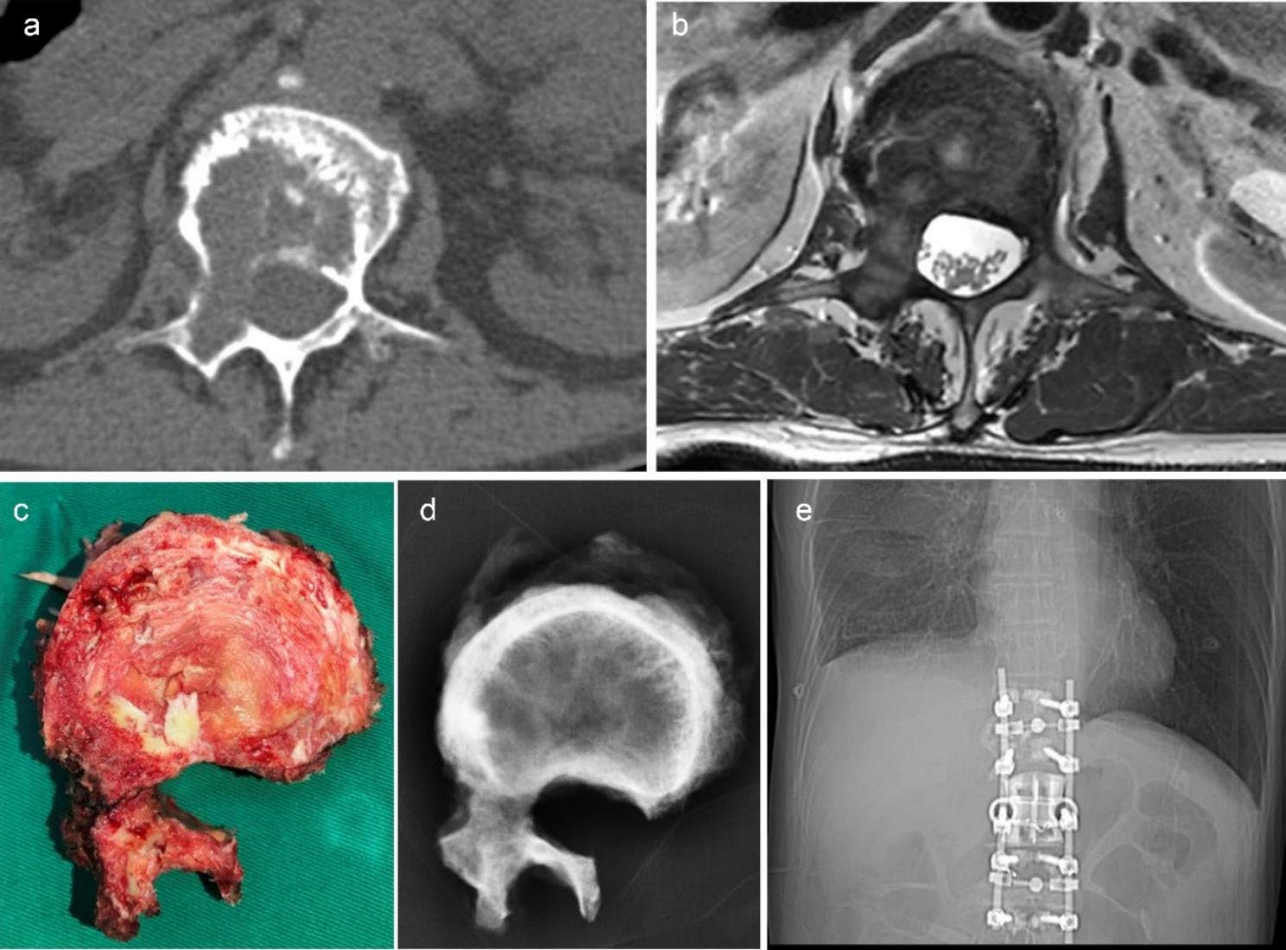
A 71-year-old male patient with L1 giant cell tumor, treated with denosumab for 1 month preoperatively, underwent WBB resection. Picture a and b were preoperative image of the tumor; picture c and d were specimen and image of the resected tumor and L11 vertebral body; picture e was postoperative image

There were three kinds of surgical procedures included: ① Total en bloc spondylectomy (TES) [8, 9]. When the pedicle is invaded by the tumor, this is effectively intralesional resection. ② En bloc resection according to the WBB surgical system (WBB resection) [10, 11]. ③ Piecemeal resection. All procedures were conducted by our surgical team. Preoperative images, surgical specimens and specimen images, and postoperative internal fixation images of TES surgery and WBB resection are shown in Figs. 1 and 2.

### Data collection

The data of these cases during hospitalization were reviewed, including demographic, imaging, clinical characteristics, and surgical data. Perioperative complications were mainly considered. According to McDonnell [18], complications were classified into major and minor depending on whether they significantly affected the patient’s recovery. The patient was reexamined in our hospital or other hospitals after surgery and regularly followed up to record local tumor control and late complications. Tumor recurrence was subject to imaging and/or pathological confirmation. All data collected and followed-up recorded by the same team.

### Statistical analysis

Categorical variables were expressed as numbers (percentages) and continuous variables as means (standard deviations). Pearson’s chi-square test or continuity-corrected chi-square test was used for categorical variables, and Fisher’s exact test was used for a small sample size. Continuous variables were analysed by one-way analysis of variance or the Kruskal‒Wallis test. SPSS 23 software (IBM, USA) was used for the above data analysis, and the significance level was set as P < 0.05.

This study was approved by the Ethical Committee of Peking university third hospital. And all methods were conducted according to the Declaration of Helsinki principles. The informed consent was waived by the Ethical Committee of Peking university third hospital because this was a retrospective study.

## Result

### Demographic and clinical characteristics

Forty-one patients were included (Table 1), consisting of 18 males (43.9%) and 23 females (56.1%), with a mean age of 34.2 years (12.6) and a mean hospital stay of 18.1 days (7.4). Thirty-one (75.6%) had thoracic vertebra lesions, and 10 (24.4%) had lumbar vertebra lesions. Thirty-five patients had primary tumors, and 6 patients had postoperative recurrence. Thirty-six patients (87.8%) had pain before surgery, and 11 patients (26.8%) had myelopathy. Preoperative neurological function was assessed by Frankel grading, with 30 patients (73.2%) classed E, 8 (19.5%) patients classed D, 2 (4.9%) patients classed C, and 1 (2.4%) patient classed A. Preoperative imaging revealed dural compression (ESCC scoring ≥ 1) in 34 cases (82.9%) and spinal cord compression (ESCC scoring ≥ 2) in 19 cases (46.3%) and indicated pedicle of vertebral arch invasion in 38 patients (92.7%). Twenty-one patients were treated with denosumab before surgery, all of whom were treated after April 2017. After this time point, only one patient did not use denosumab before surgery (sudden paraplegia, surgery as soon as possible to rescue neurological function), and the other 19 patients who accepted operation before April 2017 did not use denosumab before surgery. Two patients were treated with denosumab after operation. One patient had local recurrence, and the other was suspected of local recurrence by imaging, but the local tumor was eventually confirmed well controlled.

**Table 1:** Demographic, clinical and surgical characteristics

Variable	n
Gender, M: F	18:23
Mean age, years (sd)	34.2(12.6)
Mean hospital stay, days(sd)	18.1(7.4)
Preoperative ESCC scoring, n (%)	
0	7(17.1)
1	15(36.6)
2	6(14.6)
3	13(31.7)
Preoperative neurological function, n (%)	
Frankel A (%)	1 (2.4%)
Frankel B (%)	0
Frankel C (%)	2 (4.9%)
Frankel D (%)	8 (19.5%)
Frankel E (%)	30 (73.2%)
Location of tumor, n (%)	
Thoracic spine	31(75.6%)
Lumbar spine	10 (24.4%)
Surgical method, n (%)	
Total en bloc spondylectomy (TES) (%)	18 (43.9%)
En bloc resection by WBB surgery system (%)	12 (29.3%)
Piecemeal resection (%)	11(26.8%)
Surgical approach, n (%)	
Lateral anterior alone	1(2.4%)
Posterior alone	23 (56.1%)
Combined approach, one-stage surgery	7 (17.1%)
Combined approach, staged surgery	10(24.4%)
Affected vertebrae,n(sd)	1.32(0.69)
Resected vertebrae,n(sd)	1.71(0.90)
Preoperative embolization, n (%)	22 (53.7%)
Mean duration of surgery, min (sd)	498(176)
Mean estimated blood loss, ml (sd)	2145 (1518)

### Surgical data

Single level resection was performed in 24 cases (58.5%), and multiple level resection was performed in 17 cases (41.5%) (Table 1), with an average of 1.71 segments resected. Eighteen cases (43.9%) were treated by TES, 16 of which involved the vertebral pedicle, which was intralesional resection, and 2 cases were actual en bloc resection. Twelve patients (29.3%) underwent WBB resection, 9 of whom achieved en bloc resection, but 3 underwent intralesional resection. Eleven (26.8%) cases were performed by piecemeal resection. The lateral anterior or posterior approach alone accounted for 24 cases (58.5%). Seventeen patients (41.5%) accepted the combined approach, in which one-stage surgery accounted for 7 (17.1%). The average surgical time was 498 min (176). Twenty-two patients (53.7%) received preoperative tumor supplying artery embolization, and the mean estimated blood loss was 2145 ml (1518). Preoperative embolization reduced blood loss, but the difference was not statistically significant (1907:2422 ml, p = 0.392).

### Perioperative complications

A total of 58 complications were recorded (Table 2), with an average of 1.41 perioperative complications per patient, including 24 major complications and 34 minor complications. Thirty patients (73.2%) had at least one perioperative complication, including 15 cases with one complication, 6 cases with two complications, and 9 cases with more than three complications. Major complications occurred in 17 patients (41.5%). There were no perioperative deaths.

**Table 2:** Perioperative complications

Perioperative complications, n (%)			Treatment	Outcome
Major vascular injury, 2 (4.9%)			1: vascular suture; 1: hemostatic material compression	
Dural tear, 6 (14.6%)			All suture repair	
Pleural injury, 7 (17.1%)			6: intraoperative suture repairs; 1: drainage	
Neurological deterioration, 9 cases (22.0%)		Decreased muscle strength or hypesthesia,	Conservative treatment	All improved
CSF leakage, 6 (14.6%)			Conservative treatment	All recovered
Pleural effusion, 12 (29.3%)			Puncture drainage or closed thoracic drainage	All recovered
Respiratory infection, 3 (7.3%)			Anti-infection and oxygen inhalation	All recovered
Digestive complications, 0				
Wound-related complications, 1 (2.4%)			Debridement	Recovered
Deep venous thrombosis, 1(2.4%)			Immobilization and anticoagulation	Improved
Anemia, 3(7.3%)			Blood transfusions	All improved
Internal fixation failure, 1(2.4%)			Surgical adjustment	Recovered
Cardiovascular complications, 0				
Stroke,1 (2.4%)			Conservative treatment	Improved
Urinary complications, 3 (7.3%)	Urinary retention, 1		Indwelling catheter	All recovered
	Ureteral fistula, 1		Ureteral stenting	
	Lower urinary tract infection, 1		Anti - infection	
Chylous leakage, 1(2.4%)			Long-term indwelling drainage, nutritional support, diet control and other treatments	Recovered

#### Intraoperative complications

Two patients (4.9%) developed major vascular injury, one involving the inferior vena cava and the other involving the iliac vein. Both involved the lumbar vertebrae, with lesions reaching the anterior edge of the vertebral body. One underwent immediate vascular suturing, and the other used hemostatic material to obtain hemostasis by compression; both were satisfactory. The average blood loss and mean duration of surgery was 3500 ml (3400, 3600 ml) and 655 min (642, 667 min), which is higher and longer than other 8 lumbar surgery without major vascular injury (1563 ml, 400–2900 ml; 518 min, 274–713 min).

Dural tear occurred in 6 cases (14.6%), and pleural injury occurred in 7 cases (17.1%). Most of the patients underwent suture repair. These two intraoperative complications did not prolong the hospital stay (18.7 days).

#### Early postoperative complications

Nine patients (22.0%) had neurological deterioration after surgery, representing decreased muscle strength or hypesthesia. All patients improved by conservative treatment before discharge. Twelve patients (29.3%) presented pleural effusion requiring puncture drainage, closed thoracic drainage or long-term indwelling thoracic drainage (> 7 days). All improved after puncture or drainage. CSF leakage occurred in 6 cases (14.6%), and intraspinal tumor invasion occurred in 5 of them (WBB stage D), and 2 were recurrent cases. All improved with conservative treatment. Respiratory infection occurred in 3 cases (7.3%), and all of them recovered after anti-infection and oxygen inhalation. One patient (2.4%) underwent debridement due to poor wound healing. One patient (2.4%) was found to have lower extremity venous thrombosis after surgery, which was improved by immobilization, anticoagulation and other treatments. Three patients (7.3%) received multiple blood transfusions for anemia (≥ 2 times). One patient (2.4%) had internal fixation failure by postoperative imaging and underwent surgical adjustment within 1 week. One patient (2.4%) had intracranial hemorrhage after the operation, which improved after conservative treatment. Urinary complications occurred in 3 cases (7.3%). The most serious one was ureteral leakage after L3 surgery. A ureteral stent was placed in the urological department, and the stent was removed after improvement. One patient suffered from postoperative urinary retention, which was considered to be neurogenic bladder. The catheter was indwelling until spontaneous urination was resumed. One patient developed postoperative lower urinary tract infection, which improved after anti-infection. Postoperative chylous leakage occurred in 1 patient (2.4%), combined with pleural effusion, who improved after indwelling thoracic drainage, diet control, parenteral nutrition support.

### Follow-up

All patients were followed up after surgery for at least 2 years with no case lost. The mean follow-up time was 67 months (24–147 months), and the median follow-up time was 58 months.

#### Late complications

A total of 6 cases had postoperative internal fixation failure, all of which were titanium alloy fixation rod fractures. Three cases had one failure, and the other 3 cases had multiple internal fixation failures. All of the failures were treated with revision surgery.

#### Local tumor control

Four patients presented local recurrence (9.8%, Table 3). All of them underwent intralesional resection (4/30, 13.3%), and none had recurrence after en bloc resection (0/11). The death case was a 53-year-old female patient with L4 giant cell tumor. Local recurrence was found 3 months after piecemeal resection. After treatment with denosumab, local tumor progression was controlled. However, pain and numbness of the right lower limb and right foot drop occurred 19 months after the operation because the tumor compressed the nerve root. Therefore, palliative decompression was performed 21 months after the operation. The patient died of multiple metastasis and multiple organ failure 34 months after surgery. The mean recurrence time was 17 months (3–34 months), and the median recurrence time was 15.5 months. One patient died of tumor recurrence after surgery, and the other 3 patients were still alive.

**Table 3:** Characteristics of local recurrent cases

Case	Age/Gender	WBB stage	Surgical method	Intralesional resection	Recurrence time (m)	Treatment	Outcome
1	53 F	L4 7–12,1, A-D	Piecemeal resection	Yes	3	Denosumab, palliative operation	Died of recurrence 34 months after surgery.
2	30 F	T11 4–10 A-C	WBB resection	Yes	13	Operation was performed in another hospital	No recurrence was found 2 years after the second operation.
3	27 F	T11 2–11 A-D	TES	Yes	18	Radiotherapy	Reexamination 98 months after operation showed no obvious tumor.
4	35 M	T1-2 2–6 A-D	TES	Yes	34	Radiotherapy	Reexamination 115 months after operation showed no obvious tumor.

### Comparison of different surgical methods

TES was performed in 18 cases during 2012.5-2020.1, including 8 males and 10 females with an average age of 34.3 (19–55) (Table 4). The mean operative time was 568 min (301–909 min), and the mean blood loss was 2594 ml (900–6400 ml). Perioperative complications occurred in 15 cases (83.3%), with an average of 1.89 complications (0–4) and 0.67 major complications (0–2) per case. Sixteen patients (88.9%) underwent intralesional resection because of the tumor invading the pedicle, in which two patients (11.1%) had local recurrence.

**Table 4:** Comparison of different surgical methods

	TES, n = 18	WBB resection, n = 12	Piecemeal resection, n = 11	p
Operation time	2012.5-2020.1	2018.1-2020.7	2010.8-2018.1	
Gender, M: F	8: 10	4: 8	6: 5	0.612
Age, y (sd)	34.3(11.4)	36.0(14.5)	32.0(13.2)	0.758
Mean hospital stay, d (sd)	18.9(7.8)	14.5(4.7)	20.7(8.0)	0.105
Location of tumor, T: L	14: 4	8: 4	9: 2	0.725
Resected vertebrae, n (sd)	1.61(0.85)	1.75(0.97)	1.82(0.98)	0.858
Preoperative embolization, n (%)	8(44.4%)	9(75%)	5(45.5%)	0.232
Recurrent tumor, n (%)	4(22.2%)	0	2(18.2%)	0.209
Mean duration of surgery, min (sd)	568(184)	418(132)	484(182)	0.084
Mean estimated blood loss, ml (sd)	2594(1600)	1184(833)	2459(1589)	**0.028**
Rate of perioperative complications, n (%)	15(83.3%)	8(66.7%)	7(63.6%)	0.484
Mean number of complications, n (sd)	1.89(1.41)	1.25(1.29)	0.82(0.75)	0.118
Mean number of major complications, n (sd)	0.67(0.77)	0.83(1.03)	0.18(0.40)	0.145
Mean number of minor complications, n (sd)	1.22(0.94)	0.42(0.67)	0.64(0.67)	0.032
Intralesional resection, n (%)	16(88.9%)	3(25%)	11(100%)	**<0.001**
Local recurrence, n (%)	2(11.1%)	1(8.3%)	1(9.1%)	1.000

WBB resection was performed in 12 cases during 2018.1-2020.7, including 4 males and 8 females with an average age of 36.0 (23–71) (Table 4). The mean operative time was 418 min (253–642 min), and the mean blood loss was 1184 ml (260–3400 ml). Perioperative complications occurred in 8 cases (66.7%), with an average of 1.25 complications (0–4) and 0.83 major complications (0–3) per case. Three cases (25%) eventually underwent intralesional resection, of which 1 case (8.3%) had local recurrence.

Piecemeal resection was performed in 11 cases during 2010.8-2018.1, including 6 males and 5 females with an average age of 32.0 (11–55) (Table 4). The mean operative time was 484 min (245–711 min), and the mean blood loss was 2459 ml (1000–6000 ml). Perioperative complications occurred in 7 cases (63.6%), with an average of 0.82 complications (0–2) and 0.18 major complications (0–1) per case. All 11 cases had intralesional resection, and 1 case (9.1%) had local recurrence.

## Discussion

Complete resection is still the best treatment for this primary benign aggressive tumor [7, 19], although there are some drugs (bisphosphonates, denosumab) for treatment at present [20–25]. Both WBB resection and TES are surgical strategies to achieve en bloc resection, but in fact, they do not necessarily achieve en bloc resection. En bloc resection and intralesional resection are concepts to describe the surgical outcomes. There were also cases in our study in which en bloc resection was planned but intralesional resection was performed.

### Perioperative complications

73.2% cases had perioperative complications in our research, which was similar to that reported previously. Bandiera et al [12] and Demura et al [14] reported 50.0% and 67% complication rates after en bloc resection of spine tumors, respectively, with a large number of cases. Other studies reported complication rates ranging from 52–86.7% [15–17].

The mean blood loss was significantly less in the WBB resection group than in the other two groups. We considered three reasons for this: ① The rate of preoperative embolization in this group was the highest. ② There were no recurrent cases in this group. ③ The overall operation time of this group is the latest to date, which is affected by the improvement of experience and technology and the improvement of tools and instruments. The perioperative complication rate and average number of complications, especially the number of major complications, in the piecemeal resection group were less than those in the other two groups, suggesting that the safety of piecemeal resection might be better than that of the other two groups.

Vascular injury is most likely to occur in the large veins in front of the lumbar spine (inferior vena cava, iliac vein). The main reasons are considered, including the relatively large veins in front of the lumbar spine, the large volume of the lumbar vertebral body, the difficulty in separating the anterior structure of the vertebral body by the posterior approach, and the iliac crest interference in lower lumbar surgery. We consider that the combined approach could provide more space for tumor separation and reduce the possibility of vascular injury. Kawahara et al. [26] thought that en bloc resection of the spinal tumor in L4 or L5 could be safely achieved by posterior-anterior combined approach.

22.0% patients had neurological deterioration after surgery. For the management of nerve roots, thoracic tumors are usually directly ligated and cut off, while lumbar tumors often need to be separated and preserved. Therefore, the traction of nerve roots during lumbar surgery often leads to postoperative lower limb weakness and hypaesthesia, but the prognosis is good. Shimizu et al. [17] reported that up to 80% of patients after en bloc resection of lumbar tumors have lower limb muscle strength decline.

Pleural effusion is also a common problem, especially after thoracic tumor surgery. Transthoracic operation, resection of the pleura or chest wall tissue in order to completely remove the tumor makes the pleural cavity communicate with the surgical area and prone to pleural effusion. Prophylactic thoracic drainage is necessary in these cases.

Cerebrospinal fluid leakage are more likely to occur in intraspinal tumors and recurrent cases. The tumor is adherent to the dura mater and the boundary is not clear, which is likely to cause dural tear during separation. Yokogawa et al [27] reported that 23.6% of cases had CSF leakage after TES, and preoperative surgical site radiotherapy was a significant risk factor, which also led to adhesion.

The incidence of perioperative complications in SGCT is high, but most patients have a good prognosis after treatment.

### Local control

Although there was no significant difference in local recurrence among the three surgical methods, the local tumor control of en bloc resection was significantly better than that of intralesional resection. Compared with TES, WBB resection is more likely to achieve en bloc resection, especially in cases with pedicle involved. The purpose of piecemeal resection is only to remove the tumor completely. En bloc resection significantly reduces local recurrence of giant cell tumor of bone, which has been repeatedly demonstrated in previous studies. Luksanapruksa et al. [28] systematically reviewed the recurrence of SGCT after resection and found that 36.7% of the cases had recurrence after intralesional resection and 9.5% of the cases had recurrence after en bloc resection. Yin et al. [29] and Yokogawa et al. [30] also reported that en bloc resection was associated with a lower recurrence rate than piecemeal resection or curettage. Charest-Morin et al. [31] thought that en bloc resection should be performed when technically feasible. Our result is consistent with the results of previous studies.

Our study and previous studies have shown that en bloc resection could reduce local recurrence and prolong tumor-free survival for patients. Even with relatively high risk and many complications, en bloc resection is still necessary and worthwhile.

### Limitations

This single-center retrospective study has some limitations. It was difficult to obtain a large number of SGCT cases because of its rarity. Secondly, this study included patients treated surgically for ten years. With the development of technology and the accumulation of experience, it is difficult to ensure the consistency of treatment, and it is difficult to eliminate the influence of the above factors when comparing operations in different periods.

## Conclusion

En bloc resection is still the preferred treatment for giant cell tumors of the spine, which can reduce local recurrence. Although the surgical technique is difficult and accompanied by a high rate of perioperative complications, it can achieve favorable local control and benefit patients. When it is too difficult to complete en bloc resection, thoroughly piecemeal resection without residual is also acceptable, given the relatively low recurrence rate.

## Data Availability

The datasets used and/or analyzed during the current study available from the corresponding author on reasonable request.

## Abbreviations

GCT: Giant cell tumor
SGCT: Spinal giant cell tumor
TES: Total en bloc spondylectomy
WBB resection: En bloc resection according to WBB surgical system
CSF: Cerebrospinal fluid
